# Effects of topical vs injection treatment of cervical myofascial trigger points on headache symptoms in migraine patients: a retrospective analysis

**DOI:** 10.1186/s10194-018-0934-3

**Published:** 2018-11-08

**Authors:** Giannapia Affaitati, Raffaele Costantini, Claudio Tana, Domenico Lapenna, Cosima Schiavone, Francesco Cipollone, Maria Adele Giamberardino

**Affiliations:** 1 Headache Center, Geriatrics Clinic, Department of Medicine and Science of Aging and Ce.S.I.-Met, G. D’Annunzio University of Chieti, 66100, Chieti, Italy; 2 Institute of Surgical Pathology, G. D’Annunzio University of Chieti, Chieti, Italy; 3 Internal Medicine and Critical Subacute Care Unit, Medicine Geriatric-Rehabilitation Department, University-Hospital of Parma, Via Antonio Gramsci 14, 43126 Parma, Italy; 4 Department of Medicine and Science of Aging, G. D’Annunzio University of Chieti, Chieti, Italy; 5 Medical Clinic, G. D’Annunzio University of Chieti, Chieti, Italy

**Keywords:** Myofascial trigger points, Migraine, Nimesulide gel, Bupivacaine injection, Pain thresholds, Hyperalgesia

## Abstract

**Background:**

In migraine patients with cervical myofascial trigger points whose target areas coincide with migraine sites (M + cTrPs), TrP anesthetic injection reduces migraine symptoms, but the procedure often causes discomfort. This study evaluated if a topical TrP treatment with 3% nimesulide gel has similar efficacy as the injection but produces lesser discomfort with higher acceptability by the patients.

**Methods:**

Retrospective analysis of medical charts of M + cTrPs patients in the period January 2012–December 2016 at a single Headache Center. Three groups of 25 patients each were included, all receiving migraine prophylaxis (flunarizine 5 mg/day) for 3 months and symptomatic treatment on demand. Group 1 received no TrP treatment, group 2 received TrP injections (bupivacaine 5 mg/ml at basis, 3rd, 10th, 30th and 60th day), group 3 received daily TrP topical treatment with 1.5 g of 3% nimesulide gel for 15 consecutive days, 15 days interruption and again 15 consecutive days. The following were evaluated: monthly number of migraine attacks and rescue medications, migraine intensity; pain thresholds to skin electrical stimulation (EPTs) and muscle pressure stimulation (PPTs) in TrP and target (basis, 30th, 60th and 180th days); discomfort from, acceptability of and willingness to repeat treatment (end of study). ANOVA for repeated measures and 1-way ANOVA were used to assess temporal trends in each group and comparisons among groups, respectively. Significance level was set at *p* < 0.05.

**Results:**

Migraine improved over time in all groups, but significantly more and earlier in those receiving TrP treatment vs no TrP treatment (0.02 < *p* < 0.0001, 30–180 days for intensity and rescue medication, 60–180 days for number). All thresholds in the non-TrP-treated group did not change over time, while significantly improving in both the injection and nimesulide gel groups (0.01 < *p* < 0.0001, 30–180 days). Improvement of migraine and thresholds did not differ in the two TrP-treated groups. Discomfort was significantly lower, acceptability and willingness to repeat treatment significantly higher (0.05 < *p* < 0.0001) with gel than injection.

**Conclusion:**

In migraine patients, topical treatment of cervical TrPs with 5% nimesulide gel proves equally effective as TrP injection with local anesthetics but more acceptable by the patients. This treatment could be effectively associated to standard migraine prophylaxis to improve therapeutic outcomes.

## Background

Migraine is a frequent and highly disabling pain condition; it is the 3rd most frequent disease in the world and is classified at the 6th place, when considered alone, and at the 3rd place, when also medication-overuse is included, in the list of the most invalidating diseases worldwilde, according to the World Health Organization [1–3]. In its typical expression migraine pain is unilateral and pulsating, very intense, accompanied by nausea and/or vomiting, phono and photophobia, aggravated by physical activity; during the attack the patient most often needs to stop any activity, lying in bed, avoiding any stimuli and contact with the environment. Furthermore, a number of studies have demonstrated that, especially when the mean number of montly attacks is high (e.g., > 7/month) or the condition is chronic (> 15 headache days/month), sensitization of somatic wall tissues (skin, subcutis and muscle) may occur in the site where migraine pain is perceived, proportional in extent to the frequency of the attacks, and persisting also in between the attacks [2].

Migraine is highly comorbid with other medical conditions, most of which painful, such as fibromyalgia, visceral pain/chronic pelvic pain, as well as myofascial pain syndromes (MPS) from trigger points (TrPs), i.e., sites of exquisite tenderness located in taut, palpable bands of muscle fibers, whose stimulation produces not only local pain but also pain referred to a distant area, called target [4–14]. TrPs are very frequent in the general population and in different patient groups [15–17], but in migraine patients their prevalence is indeed significantly higher than in healthy controls [18–24]. In addition, TrPs in cervical muscles of migraineurs most often present target areas coinciding with the sites of migraine pain [9]. This specific condition of comorbidity between migraine and cervical TrPs has been shown to be responsible not only for typical myofascial pain symptoms, but also for an increase in the number and intensity of migraine attacks [11, 25]. In these patients, the sensory evaluation at TrP and target level has furthermore evidenced a pain hypersensitivity (hyperalgesia, as revealed by a decrease in pain thresholds of the superficial and deep somatic tissues) which is increased with respect to that found in patients with MPS/TrPs only or migraine only [9, 26–31]. A previous study by this group showed that TrP injection (gold standard TrP therapy) with 0.5 ml bupivacaine (5 mg/ml) in repeated sessions (n. 5, within 2 months) not only determines TrP extinction, but also produces an improvement of migraine pain [11]. Patients undergoing this treatment, in fact, present, at the end of the therapeutic cycle, a significant reduction of the mean number of monthly migraine attacks and of their intensity, together with a significant improvement of the somatic hyperalgesia at TrP and target level.

Injection therapy of TrPs, however, though representing the gold standard treatment, is not deprived of undesirable effects, mostly pain during the procedure; in addition it necessarily requires the intervention of the physician who has to perform the injection periodically [32–34]. The possibility to treat the TrP topically, e.g., with application of a Non-Steroidal-Antiinflammatory-Drug (NSAID) on the overlying skin with the ischemic-compression technique, would have the advantage of avoiding the discomfort from the injection and any potential risk linked to this invasive procedure [32]. Furthermore the patient could be instructed to apply the product autonomously and therefore carry out the therapeutic cycle at home, without the intervention of the physician. Lastly, a topical treatment could be proposed also to patients who have needle phobia and refuse TrP injection for this reason [35].

Therefore if a topical NSAID treatment of the TrP proved equally effective as the TrP injection, but with lesser side effects/discomfort it would represent a valid alternative to the injection itself in TrP treatment. Nimesulide in gel formulation possesses antiinflammatory and analgesic properties; its use has already been approved for localized musculoskeletal pain conditions such as sprains, strains and tendinopathies [36–39]. Thanks to its characteristics of hydro and liposolubility, the gel formulation guarantees optimal penetration of the active molecule through the skin into the deep parietal tissues. In addition, after topical application of the gel, systemic levels of nimesulide have been shown to be 100 times lesser than those achieved after repeated oral administration [40, 41]. This preparation therefore presents adequate characteristics to be employed in the treatment of TrPs which are typically sites of localized inflammation in muscles [42].

On this basis, in migraine patients with a high frequency of attacks the aim of the study was to investigate if repeated applications of 3% nimesulide gel over cervical TrPs with target areas coinciding with the site of migraine pain are equally effective as the TrP anesthetic injections in extinguishing the TrPs and relieving migraine symptoms while producing lesser discomfort/side effects. The study was carried out by retrospectively analyzing records of patients with migraine plus cervical TrPs who underwent TrP injection or TrP topical treatment with nimesulide gel or no TrP treatment over the same time period.

## Methods

### Patients

Charts were retrospectively reviewed of consecutive patients referred for a first visit at the Headache Center, Department of Medicine and Science of Aging of the “G. D’Annunzio” University of Chieti in the period January 2012–December 2016, diagnosed with migraine without aura and myofascial trigger points of the cervical muscles with target areas coinciding with the site of migraine pain. Based on specific inclusion/exclusion criteria, patients were divided into three groups, i.e., patients who: received no TrP treatment (group 1), received TrP treatment with local anesthetic injection (group 2), received TrP treatment with 3% nimesulide gel (group 3).

Inclusion criteria for group 1 were as follows: both sexes, age 18–65 years, a diagnosis of migraine without aura performed according to the criteria established by the International Headache Society [2, 43] at least 1 year previously; number of monthly attacks: ≥ 7 in the past 2 months; presence of one ore more active myofascial trigger points in the cervical muscles, with target area coinciding with the site of migraine pain (diagnosis performed according to Travell and Simons criteria) [32–34]; exclusion of any other headache diagnosis except migraine without aura; a history of allergy and/or intolerance to NSAIDs and/or local anesthetics and/or phobia for the use of needles, preventing topical treatment or injection of the TrP; a negative clinical history for clinical conditions known to interfere with pain sensitivity in somatic tissues of the body wall (e.g., diabetes, hypertension, fibromyalgia) [4, 5, 8, 44, 45]; absence of neurological or neuropsychiatric diseases, or cognitive deficits potentially interfering with the correct execution of the evaluations routinely performed at the Center; no prophylaxis present at the moment of the first visit; start of standard prophylaxis with flunarizine 5 mg/day carried out for 3 months from the first visit; symptomatic therapy for the attacks with paracetamol 1 g (maximal dose: 3 g/day) or paracetamol + codeine (maximal dose: 2/day) and/or triptan (maximal dose: 2 administrations/day) [46–50]; for women in their fertile phase of life, negativity of pregnancy test and use, during the whole treatment period, of validated contraceptive methods; presence of the informed consent in the patients’ records to undergo the standard evaluations and therapeutic protocols for their condition, routinely submitted to all patients at the first visit.

Inclusion criteria for patients of group 2 were the same as for group 1, except that they had a negative clinical history for intolerance to local anesthetics and presented no needle phobia, allowing local TrP treatment with anesthetic injections.

Inclusion criteria for patients of group 3 were the same as for group 1 except that they had a negative clinical history for allergy/intolerance to NSAIDs and had refused TrP injection due to either documented allergy/intolerance to local anesthetics or phobia for the use of needles, which had led to topical TrP treatment with 3% nimesulide gel.

The protocol was approved by the Institutional Review Board of the Department of Medicine and Science of Aging of the G D’Annunzio University of Chieti (Feb. 7, 2018; del. n. 90, Prot. N. 992/27.00 18, Tit III, CI 13).

Charts were reviewed consecutively, starting from December 2016 backwards, till reaching the number of 25 subjects per group.

A total of 757 charts had to be reviewed to select the 75 patients of the 3 groups.

### Treatment groups

Group 1- no TrP treatment + migraine treatment. Patients did not receive TrP treatment, but only underwent migraine treatment (prophylaxis for 3 months, symptomatic on demand).

Group 2- TrP treatment with bupivacaine injection + migraine treatment. In addition to migraine treatment, patients received TrP treatment by the medical staff at the Center with n. 5 injections of the TrP with 0.5 ml of bupivacaine (5 mg/ml) over a period of 2 months (1 injection in basal conditions and then on the 3rd, 10th, 30th and 60th day after the first visit; injection was always performed after the sensory evaluation [see below]; in the case of multiple TrPs only the most active was treated) [11]. The injection was performed according to the internationally standardized technique [17].

Group 3 - TrP treatment with topical nimesulide + migraine treatment. In addition to migraine treatment, patients received TrP topical treament with nimesulide gel. The first application was carried out at the Center by the physician, then patients were instructed to treat their TrP at home, through massage/ischemic compression, with 1.5 g (corresponding to a 3 cm strip) of 3% nimesulide gel, over the TrP for further 14 consecutive days (15 application days overall). After 15 days of interruption they had to repeat the treatment cycle for further 15 days. The technique of massage/ischemic compression involves exact detection of the TrP, application of the gel on the overlying skin, and subsequent massage of the spot by applying a moderate compression so as to determine an ischemic condition of the microcirculation and, upon release, a reactive vasodilation, with washout of algogenic substances. The compression/release cycle has to be repeated 3–4 times in succession over several minutes [32–34].

This technique, in addition to promoting the wash-out of algogenic substances, promotes a better absorption of the gel.

### Parameters examined

The following parameters are routinely evaluated at the Center in all patients with migraine at a high frequency of attacks plus cervical myofascial trigger points in basal conditions and after 30, 60 and 180 days from the start of treatment: (a) number of monthly migraine attacks through ad-hoc headache diary [10, 12, 51–54]; (b) intensity of headache attacks through numeric scale from 0 (no pain) to 10 (maximal imaginable pain) reported on the headache diary [55]; (c) monthly number of symptomatic drug consumption for the headache attacks through headache diary; (d) skin pain sensitivity through measurement of pain thresholds to electrical stimulation [EPTs] at TrP site and in the migraine pain area (target) according to a technique already described in detail elsewhere [4, 5, 10–12, 26, 35, 53, 54]; (e) muscle pain sensitivity through measurement of pain thresholds to pressure stimulation [PPTs] through Fischer’s algometer at TrP site and in the migraine pain area [56]; (f) possible occurrence of adverse events.

Only in patients undergoing TrP therapy, at the end of the evaluation period the following parameters are assessed: (g) discomfort determined by the treatment procedure of the TrP, through numerical scale from 0 (absence of discomfort) to 10 (maximal discomfort); (h) acceptability of the treatment procedure through Visual Analogue Scale (VAS) from 0 (not acceptable) to 10 (totally acceptable); (i) availability to repetition of the treatment (YES or NO).

### Technique for measurement of pain thresholds to electrical stimulation

A computerized constant current square wave electrical stimulator was used (R.S.D. stimulator, prototype, Florence 1997) [4, 5, 10, 11, 12, 26, 35, 53, 54]. The adopted stimuli were 18 msec trains of 0.5 ms square waves (internal frequency: 310 Hz), automatically delivered every 2 s.

To stimulate the skin, surface electrodes were used, constituted by 2 Ag/AgCl circular plates, 10 mm in diameter. The electrodes were positioned on the skin with interposition of a conductor paste, 1 cm apart in the longitudinal sense. Stimulation was initiated at low intensities of current (0.1 mA) and the intensity was automatically increased by the instrument, with every stimulus repeated at current increases of 0.1 mA until the subject reported a first tactile sensation and then continuing with the same rate of current increases until the subject reported a sensation of pricking pain.

Pain thresholds were measured with the method of the limits, i.e., the current value corresponding to the first report of the sensation of pricking pain was recorded and memorized by the computer, then the intensity of the stimulus was gradually decreased, always at the same rate (0.1 mA/sec), until the sensation disappeared (with recording of the corresponding current value). The intensity of the current was then again increased with recording of the corresponding value. The mean of these 3 values, automatically calculated by the stimulator computer, was considered as the final threshold for each examinated site. The subject was instructed to signal the appearance/disappearance of the sensation by pressing a button connected to the stimulator. During the whole duration of the stimulation session the subject was lying comfortably on an adjustable examination bed, in a quiet room, with the examinator close to the bed to place the electrodes and perform the test.

The subjects were informed that the evaluation test was not an endurance test for pain, that only a minimal sensation of pain had to be reported. Thery were further informed that they were free to interrupt the stimulation at any moment for any reason without any penalty.

### Technique for pain threshold measurement to pressure stimulation

The evaluation was performed through Fischer’s algometer (Great Neck, New York). The instrument is a pressure dynamometer with a rounded circular probe, 1 cm2 in diameter, with a 0-10 kg-f scale. The probe was perpendicularly positioned on the skin overlying the muscle area to be tested, and the pressure was gradually increased at a 0.1 kgf/sec rate until the subject reported a first sensation of discomfort. The corresponding kg-f value was recorded as pain threshold for that site [56].

Every subject was examined in a quiet room at constant temperature and humidity. The evaluations were always performed at the same time of day (h 9–14).

### Statistical analysis

For each group, Means ± Standard Deviation (SD) were calculated for each parameter at every evaluation time. Within each group, the temporal trend of each variable was evaluated through Analysis of Variance (ANOVA) for repeated measures. The comparison among groups, for number and intensity of attacks, and consumption of symptomatics at every evaluation time was performed through 1-way ANOVA. The comparison between the two groups of patients undergoing active TrP treatment, regarding treatment discomfort and acceptability of treatment was performed via Student’s t-test for independent data. The comparison between groups for the repeatability of treatment was performed via the chi-square test.

The level of significance was established at *p* < 0.05.

## Results

In basal conditions, the three groups proved to be homogeneous regarding sex and age, i.e., group 1 (no TrP treatment): 19 women and 6 men, 30.76 ± 8.55 years (Mean ± SD); group 2 (TrP injection treatment): 19 women and 6 men, 33.6 ± 8.21 years; group 3 (TrP nimesulide gel treatment): 18 women and 7 men, 32.64 ± 7.40 years. There were furthermore no significant differences regarding all the evaluated parameters.

Patients of all groups were affected with unilateral frontal or temporal migraine without aura (with no alternation of side) and showed myofascial TrPs in the sternocleidomastoid, semispinalis cervicis or splenius cervicis with referred pain sites (targets) coincinding with the site of migraine pain (frontal and/or temporal region).

### Migraine parameters and pain thresholds

#### Group 1 - no TrP treatment +migraine treatment

**Fig. 1:**
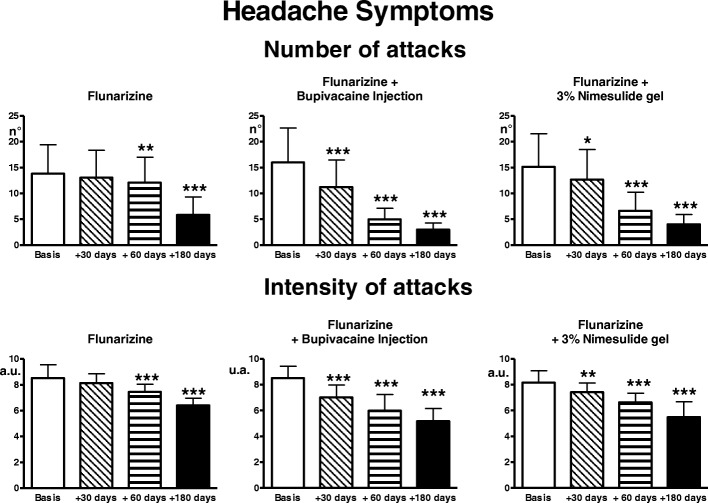
Mean monthly number of migraine attacks and their intensity recorded on the headache diary in the 3 treatment groups (flunarizine; flunarizine + bupivacaine injection of the TrP, flunarizine + 3% nimesulide gel application over the TrP)(n. 25 patients each, Means ± SD) in basal conditions (1 month preceding start of treatment) and 30, 60 and 180 days from start of treatment. * = *p* < 0.05; ** = *p* < 0.01; *** = *p* < 0.001: comparison of treatment and post-treatment evaluations with basal values

**Fig. 2:**
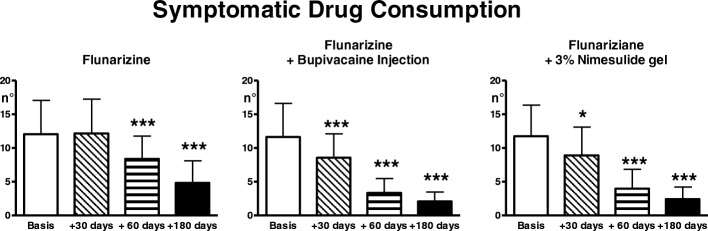
Symptomatic drug consumption for migraine attacks. Legend as for Fig. 1

Number and intensity of migraine attacks progressively decreased during the treatment period (ANOVA: *p* < 0.001), but significant effects were only evident at 60 and 180 days from the start of treatment (0.01 < *p* < 0.001) (Fig. 1). Symptomatic drug consumption also progressively decreased (ANOVA: *p* < 0.0001), with significant effects at 60 and 180 days (*p* < 0.001) (Fig. 2).

**Fig. 3:**
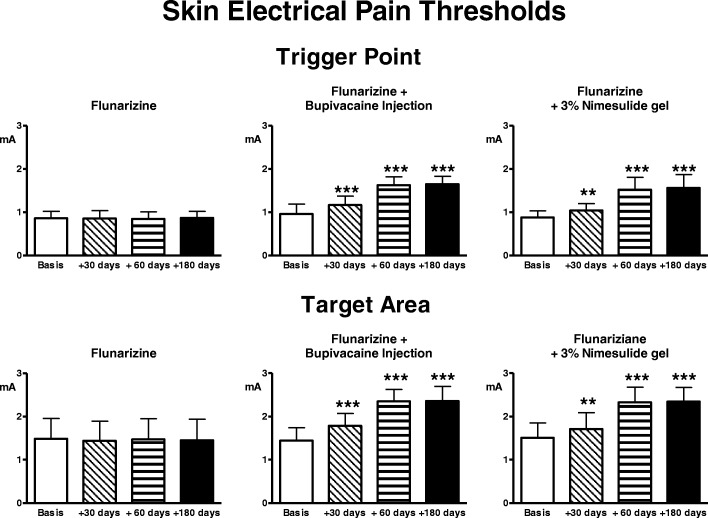
Pain thresholds to electrical stimulation of the skin overlying the trigger point and the target area. Legend as for Fig. 1

**Fig. 4:**
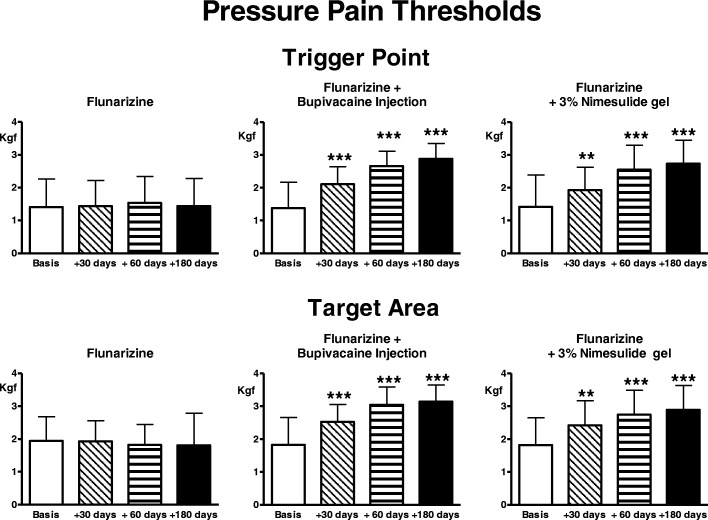
Pain thresholds to pressure stimulation in the trigger point and the target area. Legend as for Fig. 1

Pain thresholds to electrical and pressure stimulation did not undergo any significant change (Figs. 3, 4).

None of the patients reported any improvement of other concurrent pre-existing painful conditions.

#### Group 2- TrP treatment with bupivacaine injection + migraine treatment

There was a significant trend for reduction of number and intensity of migraine attacks and, in parallel, also of symptomatic drug consumption (ANOVA: *p* < 0.0001) (Figs. 1, 2). The difference was already highly significant at day 30, becoming progressively more accentuated over the study period (*p* < 0.001 for all internal comparisons).

Pain thresholds to electrical and pressure stimulation at TrP and target significantly and progressively increased with treatment (ANOVA: *p* < 0.0001), the difference was already highly significant at day 30, persisting so up to 180 days (Figs. 3, 4).

Lastly, n° 15 patients spontaneously reported an improvement of a concurrent cervicalgia/cervicobrachialgia.

#### Group 3 - TrP treatment with topical nimesulide + migraine treatment

There was a significant trend for reduction of number and intensity of migraine attacks and in parallel, also of symptomatic drug consumption (ANOVA: *p* < 0.0001) (Figs. 1, 2). The reduction was already significant at day 30 (*p* < 0.05 for number of attacks and drug consumption, *p* < 0.01 for intensity) and became progressively more accentuated over the evaluation period (*p* < 0.001 at 60 and 180 days). Pain thresholds to both electrical and pressure stimulation significantly increased with treatment in TrP and target (ANOVA: *p* < 0.0001), the difference was already significant at day 30 (*p* < 0.01) and became progressively more accentuated over the evaluation period (*p* < 0.001 at 60 and 180 days) (Figs. 3, 4).

Lastly, n. 12 patients spontaneously reported improvement of a concurrent cervicalgia/cervicobrachialgia.

### Comparison among the three patient groups

The number of migraine attacks was significantly lower in the two groups undergoing active treatment of the TrP with respect to the TrP-untreated group at 60 days (ANOVA: *p* < 0.0001, untreated vs injection and vs gel: *p* < 0.001) and 180 days (ANOVA: *p* < 0.0004, untreated vs injection: *p* < 0.001; untreated vs gel: *p* < 0.05)(Fig. 1).

The intensity of migraine attacks and the symptomatic drug consumption were significantly reduced in the two groups treated with bupivacaine injection and nimesulide gel with respect to the TrP-untreated group at 30, 60 and 180 days (for intensity at 30 days, ANOVA: *p* < 0.0001, untreated vs injection: *p* < 0.001; for intensity at 60 days, ANOVA: *p* < 0.0001, untreated vs injection: *p* < 0.001, untreated vs gel: *p* < 0.01; for intensity at 180 days, ANOVA *p* < 0.0001, untreated vs injection: *p* < 0.001; untreated vs gel: *p* < 0.01; for symptomatic drug consumption at 30 days, ANOVA: *p* < 0.02; untreated vs injection and vs gel: *p* < 0.05; at 60 days, ANOVA *p* < 0.0001, untreated vs injection and vs gel: *p* < 0.001; at 180 days, ANOVA: *p* < 0.0006, untreated vs injection: *p* < 0.001, untreated vs gel: *p* < 0.01) (Figs. 1, 2).

The comparison among groups regarding skin EPTs in the trigger showed a significant trend at 30 days (ANOVA: *p* < 0.0001, untreated vs injection: *p* < 0.001; untreated vs gel: *p* < 0.01), 60 days (ANOVA: *p* < 0.0001; untreated vs injection and vs gel: *p* < 0.001), 180 days (ANOVA: *p* < 0.0001; untreated vs injection and vs gel: *p* < 0.001).

The comparison among groups regarding skin EPTs in the target showed a significant trend at 30 days (ANOVA: *p* < 0.01, untreated vs injection: *p* < 0.05), at 60 days (ANOVA: *p* < 0.0001, untreated vs injection and vs gel: *p* < 0.001) and 180 days (ANOVA: *p* < 0.0001; untreated vs injection and vs gel: *p* < 0.001).

The comparison among groups regarding PPTs at trigger level revealed a significant trend at 30 days (ANOVA: *p* < 0.003, untreated vs injection: *p* < 0.01, untreated vs gel: *p* < 0.05), at 60 days (ANOVA: *p* < 0.0001, untreated vs injection and vs gel: *p* < 0.001) and 180 days (ANOVA: *p* < 0.0001, untreated vs injection and vs gel: *p* < 0.001).

The comparison among groups regarding PPTs in the target showed a significant trend at 30 days (ANOVA: *p* < 0.004, untreated vs injection: *p* < 0.01, untreated vs gel: *p* < 0.05), at 60 days (ANOVA: *p* < 0.0001, untreated vs injection and vs gel: *p* < 0.001) and at 180 days (ANOVA: *p* < 0.0001, untreated vs injection and vs gel: *p* < 0.001).

There was no significant difference between the two TrP active groups (bupivacaine and nimesulide) regarding migraine parameters, skin EPTs and PPTs at all evaluation times.

### End-of-study evaluation

No adverse event was recorded in any of the study groups.

**Fig. 5:**
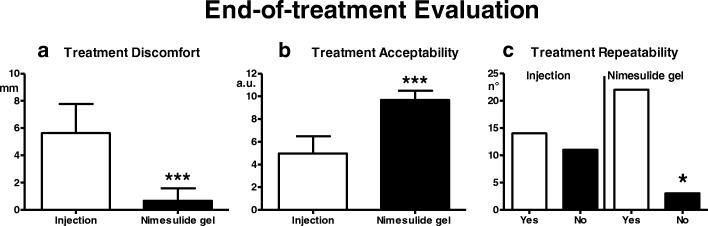
End-of-treatment evaluations. Discomfort from treatment (a), acceptability of treatment (b), willingness to repeat the treatment (c) of the TrP (injection or nimesulide gel). * = *p* < 0.05; *** = *p* < 0.001: comparison between injection group and nimesulide gel group

The discomfort from treatment in the nimesulide group was significantly lower than that recorded in the injection group (*p* < 0.0001)(Fig. 5a). For nimesulide gel, the discomfort was attributed to the residual yellow color after treatment, while in the bupivacine injection group it was mainly attributed to the pain experienced during the injection procedure.

Acceptability of the treatment was significantly higher in the nimesulide gel-treated group compared with the bupivacaine-treated group (*p* < 0.0001)(Fig. 5b).

Lastly, as regards treatment repeatability, n° 22 patients of the nimesulide group and n° 14 patients of the injection group declared they were prepared to repeat the treatment if necessary. The difference between the two groups was significant (*p* < 0.05)(Fig. 5c).

## Discussion

In migraine patients with active cervical myofascial trigger points whose target area coincides with that of migraine pain, migraine prophylaxis with flunarizine for 3 months, not associated with TrP treatment, produces a significant improvement of migraine parameters (number and intensity of attacks, symptomatic drug consumption) from the 60th day after start of treatment, and continuing at 3 months after treatment suspension (180th day) but determines no improvement in specific myofascial pain symptoms related to TrPs, as shown by the lack of any change in sensory thresholds at both TrP and target area over the same time frame.

In M + TrPs patients in whom migraine prophylaxis with flunarizine is associated with TrP treatment, either with anesthetic injection or with topical 3% nimesulide gel, the improvement of migraine parameters occurs earlier (at the 30th day) and is significantly more pronounced than that occurring in M + TrPs patients receiving migraine prophylaxis only at all determination times, until the 180th day. In addition, both TrPs treatments produce a significant improvement of the sensory asset at TrP and target level, with reduction of superficial and deep somatic hyperalgesia, which is not observed in M + TrPs patients undergoing migraine prophylaxis only. Athough the injection procedure produces slightly better results than the topical treatment at 30 days for all parameters, the difference between the two active treatments is never significant.

No prominent side effects occur with either injection or topical TrP treatment, however the former procedure produces a much higher perceived discomfort than the latter (pain at injection vs a minimal discomfort from nimesulide due to cosmetic reasons, i.e., the residual yellow coloration), as a consequence the degree of acceptability of the therapy is significantly higher for the topical treatment than for the injection. The possibility of self-administration of nimesulide gel vs necessary medical intervention for the injection is also better perceived by the patients who are therefore willing to repeat the treatment, if necessary, in a significantly higher percentage than patients subjected to the injection.

The results of the present study firstly confirm the efficacy of local treatment of cervical TrPs on migraine symptoms already shown in a previous study with anesthetic injection [11]. The pathophysiology of this phenomenon remains to be elucidated in full, but a plausible hypothesis is that suppression of the afferent sensory signals towards the Central Nervous System from the TrPs, which are powerful sources of nociceptive inputs [42], reduces the level of central sensitization of trigeminal neurons involved in migraine pain processing, thus decreasing migraine symptoms and somatic tissue hypersensitivity in the pain area [9, 11, 30].

The present study also shows for the first time that topical TrP treatment with a NSAID applied with massage/ischemic compression has similar efficacy as that of standard treatment modality with injection, but with the advantage of a better tolerability, easier modality of application and reduced costs for the health care system.

In a single Headache Center study in Italy, nimesulide orally administered was shown to represent the top preference NSAID in the treatment of acute migraine attacks [46]. Given the nonsignificant absorbtion in the general circulation of the molecule locally applied onto somatic tissues in gel formulation it is highly unlikely that the reduction of migraine symptoms in the present study, after TrP treatment with the gel, is due to a direct effect of nimesulide on the migraine condition. In addition, the duration of the benefits of the treatment goes far beyond a possible direct action of the molecule. Thus the only interpretation of migraine improvement with this treatment is an indirect action exerted by nimesulide gel onto the TrPs, similarly to what hypothesized for the effect of local anesthetic injection.

The present study has several limitations, firstly its retrospective nature, then the relatively short duration of the follow-up, and lastly the lack of a comparator group, i.e., massage/ischemic compression of the TrP without application of an active topical compound, to eliminate the confounding factor of the simple manoeuver of TrP release and separate the therapeutic effects of the manoeuver from those of the drug at local level. Further prospective studies will have to be conducted for confirmation.

In spite of these limitations, however, we believe that the results here presented have potential important therapeutic implications in migraine patients with the described characteristics.

## Conclusion

In conclusion, in migraine patients with a high frequency of attacks plus cervical myofascial trigger points with target areas coinciding with the site of migraine pain, the combination of standard migraine prophylaxis with local TrP treatment provides better efficacy results than migraine prophylaxis only. TrP topical treatment with a NSAID, such as nimesulide gel, proves equally effective as the TrP anesthetic injection, but is better tolerated by the patients, with the further advantage of the possible self-administration. These results, though preliminary, are of relevance in routine medical practice, suggesting use of this topical treatment in addition to the standard migraine prevention measures, to help enhance the therapeutic outcome [47–50, 57].

## Abbreviations

ANOVA: Analysis of Variance
EPTs: Electrical pain thresholds
M + cTrPs: Migraine patients with cervical myofascial trigger points whose target areas coincide with migraine sites
MPS: Myofascial pain syndromes
NSAID: Non Steroidal Antiinflammatory Drug
PPTs: Pressure pain thresholds
TrPs: Myofascial trigger points
VAS: Visual analogue scale
